# Rational CCL5 mutagenesis integration in a lactobacilli platform generates extremely potent HIV-1 blockers

**DOI:** 10.1038/s41598-018-20300-9

**Published:** 2018-01-30

**Authors:** Massimiliano Secchi, Valentina Grampa, Luca Vangelista

**Affiliations:** 1 Protein Engineering and Therapeutics Group, Division of Immunology, Transplantation and Infectious Diseases, San Raffaele Scientific Institute, 20132 Milan, Italy; 2 Department of Biomedical Sciences, School of Medicine, Nazarbayev University, 010000 Astana, Kazakhstan; 3 Present Address: INSERM, UMRS-839, Institut du Fer à Moulin, 75005 Paris, France

## Abstract

Efforts to improve existing anti-HIV-1 therapies or develop preventatives have identified CCR5 as an important target and CCL5 as an ideal scaffold to sculpt potent HIV-1 entry inhibitors. We created novel human CCL5 variants that exhibit exceptional anti-HIV-1 features using recombinant lactobacilli (exploited for live microbicide development) as a screening platform. Protein design, expression and anti-HIV-1 activity flowed in iterative cycles, with a stepwise integration of successful mutations and refinement of an initial CCL5 mutant battery towards the generation of two ultimate CCL5 derivatives, a CCR5 agonist and a CCR5 antagonist with similar anti-HIV-1 potency. The CCR5 antagonist was tested in human macrophages and against primary R5 HIV-1 strains, exhibiting cross-clade low picomolar IC_50_ activity. Moreover, its successful combination with several HIV-1 inhibitors provided the ground for conceiving therapeutic and preventative anti-HIV-1 cocktails. Beyond HIV-1 infection, these CCL5 derivatives may now be tested against several inflammation-related pathologies where the CCL5:CCR5 axis plays a relevant role.

## Introduction

HIV-1 entry into the target cell is a complex series of molecular events involving several protein players. This starts from virus docking and, through several protein-protein interactions and major conformational changes, ends with virus-cell membrane fusion. The complexity of HIV-1 architecture and entry dynamics reflects the difficulties encountered so far in the development of an efficacious vaccine^1,2^. CCR5 and CXCR4 represent the major HIV-1 co-receptors, however CXCR4 tropism insurgence occurs during HIV-1 infection, while CCR5 is the most exclusively used co-receptor in primary infections. In this molecular scenario, HIV-1 gp120 and cellular CCR5 are crucial entities that represent strategic targets for anti-HIV-1 therapeutic and preventative drug development^3^. Interest towards CCR5 as an anti-HIV-1 target has been steadily growing, with maraviroc (MVC), a small chemical compound, currently being used systemically and tested for topical prevention^4^. CCL5/RANTES, a natural ligand of CCR5 and a potent HIV-1 entry inhibitor, is an anti-HIV-1 lead and a very important protein alternative to CCR5-targeting small chemical compounds^5,6^. With the identification of CCL5, CCL3/MIP-1α, CCL4/MIP-1β and CXCL12/SDF-1 as natural HIV-1 inhibitors and of CCR5 and CXCR4 as HIV-1 co-receptors, a totally new view on the chemokine system provided investigators with novel targets to combat HIV-1 cell entry and infection^7^. Given its anti-HIV-1 potency and its extensive structural characterization, human CCL5 is an ideal molecular template for the engineering of anti-HIV-1 CCR5 antagonist variants. In a therapeutic or prophylactic regimen, chronic activation of CCR5 could promote undesirable inflammatory effects, thus CCR5 antagonism is seen as a necessary requisite. However, a wealth of powerful CCL5 derivatives acting as CCR5 agonists have been produced, with PSC-RANTES being the most potent anti-HIV-1 variant to date^8^. The chemical modification at its N-terminus represents a drawback for PSC-RANTES, as it does not allow its expression as recombinant protein. Considering the needs for CCR5 antagonism, the possibility of expression in recombinant systems, a high anti-HIV-1 potency and the implementation as anti-HIV-1 topical microbicide, a CCL5 mutant recapitulating all these features has been developed, C1C5 RANTES^9–11^. Subsequently, a superior variant was produced, 5p12-RANTES, that blocks HIV-1 with potency comparable to PSC-RANTES, yet acting as CCR5 antagonist and suitable to recombinant expression^12^.

Within the field of drug and system development for the prevention of HIV-1 infection, topical microbicides represent an alternative and a complementary option to vaccines^13–15^. Live microbicides are based on the engineering of commensal bacteria to deliver anti-HIV-1 agents *in vivo* and *in situ*^16,17^. Commensal bacteria delivery of protein therapeutics conveys a wide interest for several pathologies^18^.

This work stems from the reported production of wild type (wt) CCL5 and C1C5 RANTES^10,11^ and deals with the use of commensal lactobacilli as a platform for the screening of conceptually novel CCL5 mutants, embracing efficient CCL5 secretion, low cost, easy handling and suitability to live microbicide development. CCL5 expression has been tested in two *Lactobacillus* strains, providing proof of principle for vaginal and intestinal applicability. CCL5 mutant design and selection yielded a CCR5 agonist with a native N-terminus (CCL5 5 m) that presents anti-HIV-1 potency comparable to PSC-RANTES and 6p4-RANTES (a potent CCR5 agonist CCL5 variant)^12^. The five mutations selected and incorporated in CCL5 5 m were inserted in CCL5 variants presenting the 5p12 and 6p4 N-terminus (CCL5 5p12 5 m and CCL5 6p4 5 m), yielding a five-fold anti-HIV-1 potency increase over 5p12-RANTES and 6p4-RANTES. The pharmaceutical sector is now provided with a series of extremely potent CCL5 variants apt to development as HIV-1 blockers, potential anti-inflammatory agents and lead compounds for those pathologies where CCL5 is of major relevance^19,20^.

## Results and Discussion

### The lactobacilli platform

**Figure 1:**
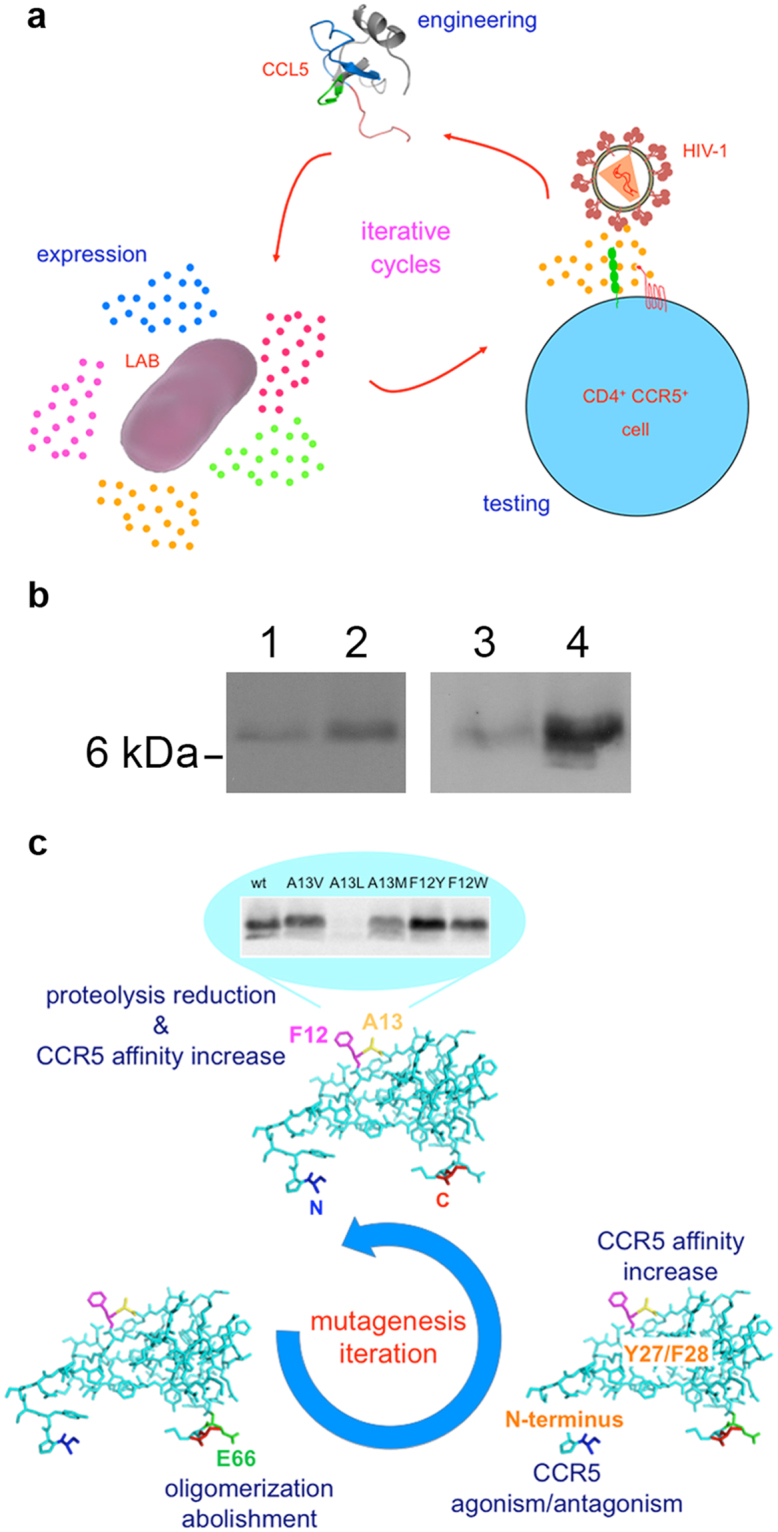
Expression of CCL5 mutants in lactobacilli. (**a**) LAB as a platform to screen novel CCL5 mutants *via* iterative cycles of gain of function consisting of CCL5 engineering, expression and anti-HIV-1 activity testing. Colored dots represent different CCL5 mutants. (**b**) Expression of wt CCL5 and C1C5 RANTES in *L*. *rhamnosus* GG as detected in Western blot. Codon-optimized C1C5 RANTES (lane 1), codon-optimized wt CCL5 (lane 2) (the gel image for lanes 1 and 2 was cropped to improve clarity, see complete gel in Supplementary Figure S2), non codon-optimized C1C5 RANTES (lane 3), codon-optimized C1C5 RANTES (lane 4) (the gel image for lanes 3 and 4 was cropped to improve clarity, see complete gel in Supplementary Figure S3). (**c**) Schematic representation of the CCL5 hotspots targeted to achieve: CCR5 antagonism (N-terminus), abolishment of oligomerization (E66), CCR5 affinity increase (amino acid F27 and Y28) and CCL5 proteolysis reduction and CCR5 affinity increase (amino acid F12 and A13). A representative Western blot of CCL5 mutants at position 12 and 13 is illustrated (the gel image was cropped to improve clarity, see complete gel in Supplementary Figure S4). N and C, N- and C-terminus.

Lactic acid bacteria (LAB) present several interesting features that make them very attractive in biomedicine and provide many advantages for public health. Being part of the human microbiome with the status of GRAS (generally regarded as safe), recombinant LAB have been identified as an optimal system for the live delivery of protein therapeutics^18^. Many different expression systems have been previously used for CCL5 engineering and production, the most common being *E*. *coli*. Lactobacilli were excellent recombinant protein secretors as they could produce and fold a rather difficult protein such as C1C5 RANTES (in which S1 and S5 have been mutated to C) in a superior manner as compared to mammalian cells^9^. The features mentioned above have been combined here in a system devised to finely select conceptually novel CCL5 mutants. Codon-optimized CCL5^10^ was engineered in a lactobacilli-dedicated plasmid, followed by cycles of expression in the vaginal strain *L*. *jensenii*, semi-purification and anti-HIV-1 testing (acute infection assays) of the secreted CCL5 mutants (Fig. 1a). Codon-optimized wt CCL5 and C1C5 RANTES secretion was tested also in the intestinal strain *L*. *rhamnosus GG* (437 and 226 μg/l, respectively), with a significant improvement in the secretion level upon codon optimization of C1C5 RANTES (non codon-optimized C1C5 RANTES 83 μg/l) (Fig. 1b). The successful expression of CCL5 in an intestinal *Lactobacillus* strain provides an interesting option on the use of CCL5 as intestinal live anti-HIV-1 microbicide and anti-inflammatory agent.

### Division of CCL5 into CCR5-dedicated hotspots

In order to produce and screen novel mutants with potent anti-HIV-1 activity, CCL5 has been tackled on different hotspots aiming at the achievement of a CCR5 interaction enhancement. Hotspots were targeted to obtain four distinct changes/enhancements in the way CCL5 interacts with CCR5. These were: i) conversion of CCL5 into a CCR5 antagonist; ii) abolishment/reduction of CCL5 oligomerization; iii) affinity increase in CCL5 binding to CCR5; and iv) reduction of lactobacilli proteolysis towards recombinant CCL5. Each conceptual point corresponded to the targeting of a defined region of CCL5 with successful mutations being integrated through iterative cycles of mutant characterization (Fig. 1a,c).

### Conversion of CCL5 into CCR5 antagonist and potent agonist variants

A major requirement for the use of CCL5 derivatives as anti-HIV-1 blockers is the conversion of CCL5, by N-terminus modification, into a CCR5 antagonist. Four different CCR5 antagonists have been implemented in this study, three were drawn from previous reports (C1C5 RANTES, L-RANTES and 5p12-RANTES)^9–12^ and one is a new attempt to convert CCL5 into an antagonist, CCL7/CCL5, a chimera in which the first 10 N-terminal amino acids of CCL7, a natural CCR5 antagonist, were placed before CCL5 C10 (CCL7 has an homologous cysteine residue at position 11)^21^. The production of 6p4-RANTES, a potent CCR5 agonist^12^ was also attempted. The level of secretion of these CCL5 variants differed significantly, with 5p12-RANTES and 6p4-RANTES presenting the most dramatic reduction (Table 1). In its progression, the selection process was accomplished by decreasing the number of mutants, eliminating those presenting the lowest antiviral activity and secretion level.

**Table 1:** *L*. *jensenii* CCL5 mutants expression and anti HIV-1 activity.

Mutant	Expression (ng/ml)	IC_50_ (pM)
wt CCL5	657	540
CCL5 E66S	450	190
CCL5 T7K	296	822
CCL5 T7K/E66S	282	813
CCL5 Y27W	172	255
CCL5 F28W	150	162
CCL5 Y27W/F28W	52	95
CCL5 Y27W/F28W/E66S	28.3	46
CCL5 F12W	948	>6500
CCL5 F12Y	1341	311
CCL5 F12P	228	>1000
CCL5 A13V	240	86
CCL5 A13L	5	ND
CCL5 A13M	204	256
CCL5 A13P	20	>1000
CCL5 A13F	5	92
CCL5 A13Y	55	78
CCL5 A13W	51	169
CCL5 F12Y/A13V	150	179
CCL5 F12Y/A13V/Y27W/F28W (CCL5 4 m)	20	115
CCL5 F12Y/A13V/Y27W/F28W/E66S (CCL5 5 m)	20	34
C1C5 RANTES	438	5000
C1C5 E66S	156	1600
C1C5 Y27W	58	3700
C1C5 27W/28 W	20	833
C1C5 Y27W/F28W/E66S	14	640
L-RANTES	57	6400
L E66S	49	2100
L Y27W	25	2500
L Y27W/F28W	1.7	1060
L Y27W/F28W/E66S	1.6	787
CCL7/CCL5	123	9000
CCL7/CCL5 E66S	36	3000
CCL7/CCL5 Y27W	18	3600
CCL7/CCL5 Y27W/F28W	1	1500
CCL7/CCL5 Y27W/F28W/E66S	1.5	1100
5p12-RANTES	0.8	29
5p12 E66S	2.4	18
5p12 Y27W	0.2	17
5p12 Y27W/F28W	0.2	8.2
5p12 Y27W/F28W/E66S	0.1	7.7
5p12 F12Y/A13V	1	30
5p12 F12Y/A13V/Y27W/F28W/E66S (CCL5 5p12 5 m)	0.1	5.5
6p4-RANTES	0.2	30
6p4 F12Y/A13V/Y27W/F28W/E66S (CCL5 6p4 5 m)	0.05	4.7

ND, not determined

### Abolishment of CCL5 oligomerization

Two substitutions were introduced in order to abolish CCL5 oligomerization: the well-known E66S^22^ and the T7K mutation. Glutamic acid residue E66 is responsible for the oligomerization of CCL5 *via* electrostatic interactions. Hence, the elimination of E66 negative charge by its mutation to serine prevents CCL5 oligomerization and resolves the CCL5 solution state to a simple monomer-dimer equilibrium, resulting in a higher number of CCL5 monomers available in solution. As a reflection, CCL5 E66S presents a higher anti-HIV-1 activity as compared to wt CCL5 (Table 1). This is an interesting case of gain of function, since the increase in antiviral activity is most likely not attributable to residues E66 or E66S in their interaction with CCR5, rather it is the reflection of the increased number of monomers available in the E66S mutant. Indeed, CCL5 appears to interact with CCR5 in its monomeric form^23^, although the dimer seems to participate in a weaker manner^24^, thus an increase in the number of CCL5 monomers available in solution should lead to the observed increase of anti-HIV-1 activity. Interestingly, a 5p12-RANTES-E66S variant has been generated and characterized by NMR to be exclusively monomeric in solution^25^. A role has been hypothesized for residue T7 in CCL5 dimerization, hence the T7K mutation was introduced as a mean to provide a fully monomeric CCL5, *e*.*g*., with the CCL5 T7K/E66S mutant. However, the T7K mutation within both CCL5 T7K and CCL5 T7K/E66S decreases CCL5 anti-HIV-1 activity (Table 1), possibly due to a direct interaction of residue T7 with CCR5 in monomeric CCL5. This experimental evidence for T7 participation to CCR5 binding has also been proposed in a three-dimensional model of the CCL5-CCR5 interaction^26^. Clearly, this result thwarted the use of the T7K mutation to improve CCL5 antiviral features. We therefore kept the T7 residue in wt, C1C5, L, CCL7 (homologous T) and 5p12 variants, while that residue is not present in the 6p4 variant.

### CCL5 increase of CCR5 affinity

Rational design of CCL5-based CCR5 inhibitors is hampered by the lack of detailed three-dimensional structure information on the CCL5-CCR5 interaction. However, the three-dimensional structure determination of CCR5 in complex with MVC^27^ and a structural modeling of CCL5-CCR5 interaction^26^ shed new light in the docking surfaces involved in this important protein-protein interaction. Yet, several CCL5 features have been investigated in this direction by alternative means. Studies on CCL5-derived peptides identified the N-loop/β1-strand as the region responsible for the docking of the protein onto CCR5^28^ and two hydrophobic patches spanning amino acids at position 11–15 and 27–29 as those crucial for receptor interaction^29^. Attempts to increase CCL5-derived peptides anti-HIV-1 potency by substitutions of F12 were frustrated by the need to maintain the original amino acid. However, modification of F28 with a non natural amino acid^30^ and the subsequent modification of Y27 and F28 with natural W residues^31^ highlighted the possibility to increase the activity of CCL5-derived peptides. We then adopted here the WW substitution at CCL5 positions 27 and 28 in the direction of an affinity increase towards CCR5. An initial Y27W substitution was introduced in wt, C1C5, L, CCL7 and 5p12 N-terminal variants, followed by single F28W and double Y27W/F28W mutations (Table 1). In all cases, an increase in anti-HIV-1 activity was observed with a concomitant decrease in the protein secretion level (Table 1). A further integration was accomplished by adding the E66S mutation, again witnessing an increase in antiviral potency (Table 1). At this stage, given their significantly lower antiviral activity, the CCL5 variants presenting the C1C5, L and CCL7 N-terminus were abandoned and the wt and 5p12 variants were carried over as lead CCR5 agonist and antagonist variants.

### Simultaneous proteolysis reduction and CCR5 binding improvement

Despite the numerous advantages provided by lactobacilli as an expression system and a live microbicide platform, a significant drawback is represented by the frequent proteolytic cleavage encountered upon recombinant protein expression. CCL5 has not been spared by this phenomenon and a proteolytic cleavage has been identified that affects about 40% of the total protein secreted by lactobacilli, with the cleavage site occurring between amino acids in position 12 and 13^10,11^. Besides the proteolytic cleavage by lactobacilli, a debated sensitivity to proteolysis has been reported also for PSC-RANTES^32^. Residues in position 12 and 13 have been identified as crucial players in the interaction of CCL5 with CCR5. As mentioned earlier, this knowledge has been acquired during the studies on CCL5-derived peptides. Substitution of amino acids in position 12 and 13 could thus simultaneously serve two needs, the reduction of proteolysis and an increase in the antiviral activity. We then proceeded with a series of mutants (using wt CCL5 as scaffold) at both positions: F12 (mutated to W, Y and P), and A13 (mutated to W, Y, M, F, V, L and P). These residues were selected based on the need to preserve the hydrophobic nature of the amino acid side chain, as this is likely to be the requirement for an increase in the CCR5 interaction. As mentioned above, F12 substitutions within CCL5-derived short peptides did not yield any antiviral improvement^29^. Indeed, in the context of the full-length protein, the F12W mutant provided a very good improvement in the secretion level and proteolysis reduction, yet it presented a dramatic decrease in the anti-HIV-1 activity. Even though the F12P mutation totally eliminated the proteolytic cleavage, this important effect was accompanied by a decrease in the secretion level and antiviral activity. Conversely, a striking concomitant improvement in the secretion level (two-fold compared to wt CCL5), antiviral activity and proteolysis reduction was observed when testing the F12Y, confirming this position as a crucial hotspot for CCR5 binding, yet confuting all previous assumptions for the exclusive importance of residue F12 (Table 1 and Fig. 1c). Concerning the A13 mutation, all residues tested led to a decrease in the secretion level, however residues W, Y, M, F, and V presented an increase in antiviral potency (Table 1). Concerning the proteolysis reduction, the A13L mutant presented a nearly complete cleavage that hampered the antiviral activity determination. The A13M mutation led to a proteolysis increase, while the A13P mutant totally abolished the proteolysis, yet with a decrease in antiviral activity. The A13V, A13Y and A13F presented a similar decrease in proteolytic cleavage as well as a similar increase in antiviral activity, however the A13V mutant provided the best secretion level. The A13W mutant eliminated the proteolytic cleavage, however the secretion level and antiviral activity were lower than those of A13V (Table 1). Overall, the CCL5 A13V mutant presented the best features within the A13 mutant battery, *i*.*e*., secretion level, antiviral activity and proteolysis reduction. When introduced in CCL5-derived peptides, the A13P substitution provided a gain in antiviral activity and the A13V substitution resulted in a significant reduction of the peptide antiviral potency^30^. The discrepancy with the full-length mutant counterparts attests that the molecular environment and folding options are often different within a short peptide or a full-length protein. The double F12Y/A13V mutants were then engineered within the wt and 5p12 N-terminal CCL5 variants, resulting in secretion levels and anti-HIV-1 activities slightly dissatisfying the expectations. In other words, in comparison to wt CCL5, the wished additive or even synergic effect of coupling the two successful mutations at positions 12 and 13 led to an activity increase for CCL5 F12Y/A13V lower than that obtained with CCL5 A13V. The antiviral activity and secretion level for CCL5 5p12 F12Y/A13V were comparable to that of 5p12-RANTES, while the level of secretion was slightly decreased for CCL5 F12Y/A13V *vs* wt CCL5. This less than optimal behavior might be attributable to a certain degree of steric hindrance in the contiguous FA *vs* YV couples or in the surrounding microenvironment. Nevertheless, the double F12Y/A13V mutation was adopted in light of the proteolysis reduction and increase in antiviral potency.

### CCL5 hotspots mutagenesis integration

Finally, we created a series of CCL5 mutants recapitulating and incorporating all successful mutations (see Supplementary Table S1 recapitulating all mutants generated in this work). Compared to wt CCL5, the CCL5 F12Y/A13V/Y27W/F28W mutant (named CCL5 4 m for simplicity) presented a five-fold increase in anti-HIV-1 activity and CCL5 4 m with the extra E66S mutation (named CCL5 5 m) led to a further three-fold antiviral activity increase over CCL5 4 m, reaching a potency comparable to 6p4-RANTES, yet with an unmodified N-terminus (see Supplementary Figure S1 for a linear sequence representation of wt CCL5 and CCL5 5 m). The CCL5 5p12 F12Y/A13V/Y27W/F28W/E66S mutant (named CCL5 5p12 5 m) led to a five-fold activity increase over 5p12-RANTES. The 6p4 N-terminal variant was rescued after an initial exclusion due to its very low expression level in *L*. *jensenii*. Hence, the five successful mutations (5 m) led to CCL5 6p4 5 m that presented also a five-fold anti-HIV-1 activity increase over 6p4-RANTES (Table 1).

The 6p4 and wt N-terminal variants incorporating all the successful mutations constitute lead compounds exploitable in those pathologies, other than HIV-1 infection, where potent CCR5 agonists could be desirable (*e*.*g*., antitumor adjuvanticity)^33^.

### CCL5 mutants expression in *E*. *coli*

**Figure 2:**
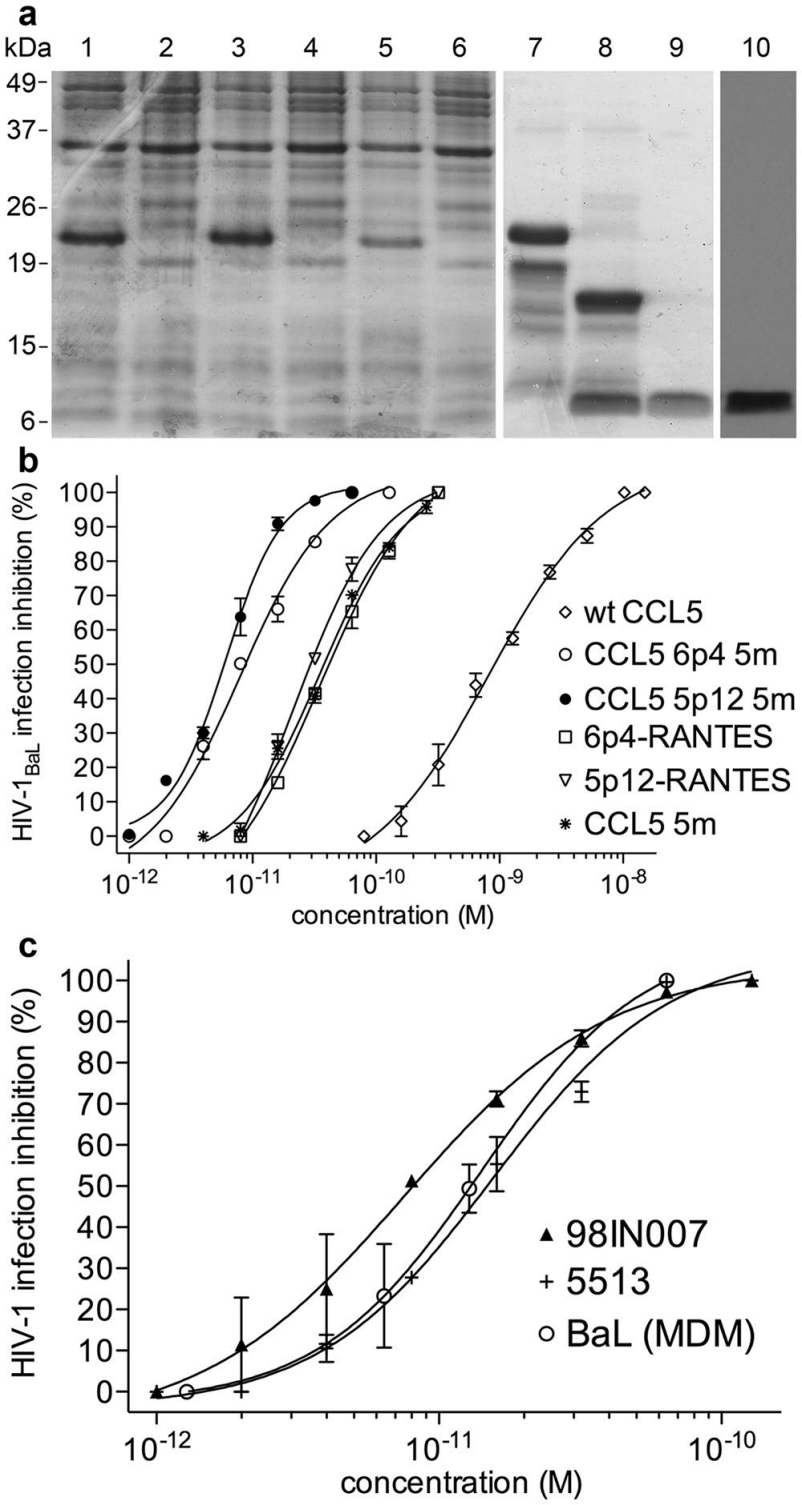
Anti-HIV-1 activity of CCL5 mutants purified from *E*. *coli*. (**a**) SDS-PAGE stained with Coomassie blue of *E*.*coli* total lysates expressing CCL5 (lanes 1 and 2), CCL5 5 m (lanes 3 and 4) and 5p12-RANTES (lanes 5 and 6). Lanes 1, 3 and 5 were induced with 1 mM IPTG. Expressed proteins migrate with an expected molecular mass of 21 kDa. After purification and digestion with SUMO protease CCL5 variants migrated with a molecular mass of 7.8 kDa, as illustrated for CCL5 5p12 5 m in SDS-PAGE stained with Coomassie blue (lanes 7–9) and Western blot (lane 10). SUMO-CCL5 5p12 5 m (lane 7), CCL5 5p12 5 m digested with ULP1 (lane 8), purified CCL5 5p12 5 m (lanes 9 and 10) (the gel images for lanes 1 to 6, 7 to 9 and 10 were cropped to improve clarity, see complete gels in Supplementary Figures S5–7, respectively). (**b**) HIV-1_BaL_ inhibition by our best CCL5 derivatives tested by acute infection assays in PM1 cells and measured by a p24-based assay after 4 days of infection. Values indicate the means ± SD of two independent experiments performed in triplicate. (**c**) HIV-1 inhibition of CCL5 5p12 5 m tested by acute infection assay as in Fig. 2b in PM1 cells against the primary HIV-1 strains 5513 and 98IN007 and in MDM against the laboratory strain BaL. Values indicate the means ± SD of two independent experiments performed in triplicate.

In order to obtain higher amounts of CCL5 mutants for their further characterization, wt CCL5, CCL5 5 m, 5p12-RANTES, CCL5 5p12 5 m, 6p4-RANTES and CCL5 6p4 5 m were cloned into the pET SUMO plasmid and expressed in *E*. *coli* as recombinant proteins N-terminally fused to the SUMO tag, as already reported for 5p12-RANTES^34^. As expected, the SUMO-fused CCL5 variants presented an apparent molecular mass of 21 kDa in Coomassie blue-stained SDS-PAGE (Fig. 2a) and their expression levels are summarize in Table 2, showing that the shift from *L*. *jensenii* to the *E*.*coli* SUMO expression system was very efficient. While the production of recombinant wt CCL5 was similar in both systems (~0.5 mg/l), the expression level of *E*. *coli*-produced CCL5-mutants was ~ 0.1–0.5 mg/l, with an increase from 25 up to 2000 folds (Tables 1 and 2). Next, the CCL5 mutants were purified to homogeneity and SUMO tags were removed using the ULP1 SUMO protease^35^ to obtain the expected 7.8 kDa CCL5 molecular mass. As shown by Coomassie blue staining and Western blot for CCL5 5p12 5 m, CCL5 variants were purified after SUMO cleavage (Fig. 2a). Purified CCL5 mutants were tested in acute infection assays using HIV-1_BaL_ (Fig. 2b). The IC_50_s (Table 2) were similar to those obtained with *L*. *jensenii*-produced semi-purified proteins (Table 1), confirming that the activity of CCL5 5 m is comparable to that of 6p4-RANTES (IC_50_ 35 pM) and that the introduction of the five strategic mutations in the scaffold of 5p12-RANTES and 6p4-RANTES generated a superior anti-HIV-1 CCR5 antagonist (CCL5 5p12 5 m, IC_50_ 6.1 pM) and agonist (CCL5 6p4 5 m, IC_50_ 7.9 pM), respectively. Moreover, purified CCL5 5p12 5 m inhibited HIV-1_BaL_ infection in human monocyte-derived macrophages (MDM) with an IC_50_ of 14.2 pM (Fig. 2c). The strong anti-HIV-1 activity of CCL5 5p12 5 m was further confirmed by the inhibition of two primary R5 isolates in PM1 cells. The HIV-1 strains 5513 (clade B) and 98IN007 (clade C) were inhibited in acute infection assay with an IC_50_ of 15.5 and 7.6 pM, respectively (Fig. 2c).

**Table 2:** *E*. *coli* CCL5 mutants expression and anti HIV-1 activity.

CCL5 mutant	Expression (mg/l)	IC_50_ (pM)
wt CCL5	0.5	810
CCL5 5 m	0.5	35
5p12-RANTES	0.1	21
CCL5 5p12 5 m	0.1	6.1
6p4-RANTES	0.1	35
CCL5 6p4 5 m	0.1	7.9

### CCL5 5p12 5 m acts in synergy with several anti-HIV-1 compounds

**Figure 3:**
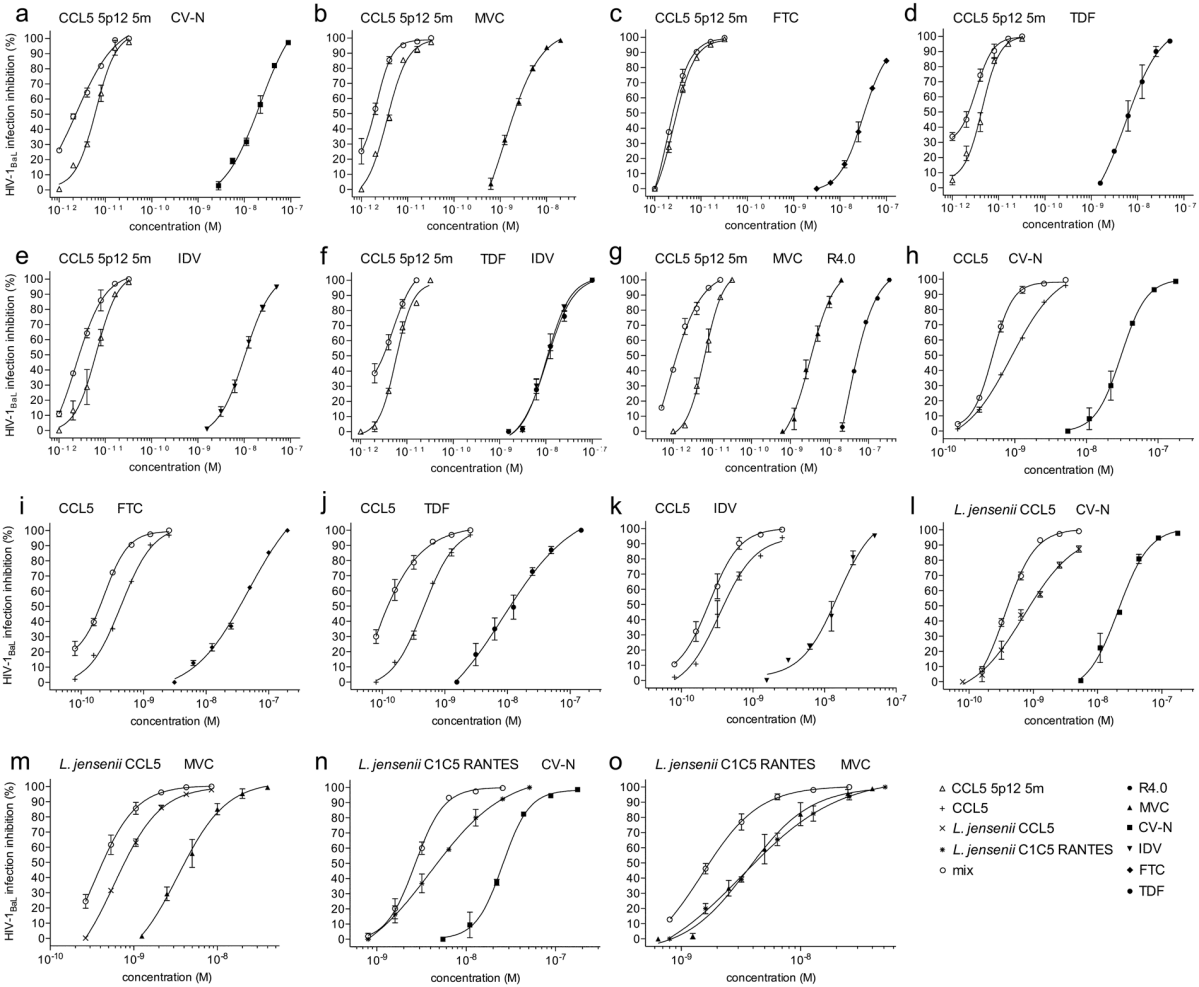
Anti-HIV-1 activity of CCL5 5p12 5 m, wt CCL5 and C1C5 RANTES in combination with HIV-1 inhibitors of different classes. (**a–o**) HIV-1_BaL_ inhibition was evaluated by acute infection assays as described in Fig. 2b. *E*. *coli* recombinant CCL5 5p12 5 m was tested in combination with CV-N (**a**), MVC (**b**), FTC (**c**), TDF (**d**), IDV (**e**) and in triple combination with TDF and IDV (**f**) and with MVC and R4.0 (**g**). *E*. *coli* recombinant wt CCL5 was tested in combination with CV-N (**h**), FTC (**i**), TDF (**j**) and IDV (**k**). *L*. *jensenii* recombinant wt CCL5 was tested in combination with CV-N (**l**) and MVC (**m**). *L*. *jensenii* recombinant C1C5 RANTES was tested in combination with CV-N (**N**) and MVC (**o**). The dose-response curves of mixed inhibitors (mix) are referred to CCL5 5p12 5 m (**a–g**), wt CCL5 (**h–m**) and C1C5 RANTES (**N**,**O**) (p-values < 0.0001). Values indicate the means ± SD of two independent experiments performed in triplicate.

Based on HIV-1/AIDS treatment regimens or microbicide cocktails that combine antiretroviral compounds of different classes to maximize their protective effect^36^, we investigated the potential of CCL5 5p12 5 m in combination with different anti-HIV-1 drugs and lead compounds that target different stages of HIV-1 infection. Tested in acute infection assays against HIV-1_BaL_ (Fig. 3a–e), the anti-HIV-1 compounds used in combination with CCL5 5p12 5 m were: cyanovirin-N (CV-N), that binds the carbohydrate shield of gp120^37,38^; MVC, a CCR5 antagonist^4^; emtricitabine (FTC) and tenofovir (TDF), two reverse transcriptase inhibitors^39,40^; and indinavir (IDV), a protease inhibitor^41^. Although less potent than new protease inhibitors, IDV has been used here as a proof of concept. As illustrated with the dose-response curves of mixed inhibitors (Fig. 3), the IC_50_ of CCL5 5p12 5 m improved in all combinations tested from 1.3 to 2.9 folds. The calculated combination indexes summarized in Table 3 confirmed that all the inhibitors synergized with CCL5 5p12 5 m, yielding combination index (CI_50_) values ranging from 0.72 to 0.89. CCL5 5p12 5 m inhibition of HIV-1_BaL_ infection was also evaluated in the triple combinations CCL5 5p12 5 m:TDF:IDV and CCL5 5p12 5 m:MVC:R4.0 (Fig. 3f,g). The possibility to successfully target different steps of HIV-1 infection or to simultaneously target CCR5 with three different CCR5 entry inhibitors has been explored with MVC, CCL5 and the CCL5-derived peptide R4.0 and discussed thoroughly^42^. Both triple combinations resulted in an additive effect with a CI_50_ of 1.16 for CCL5 5p12 5 m:TDF:IDV and a CI_50_ of 0.9 for CCL5 5p12 5 m:MVC:R4.0, improving the IC_50_ of CCL5 5p12 5 m of 1.3 and 9.1 folds, respectively (Table 3). As a control, a synergic or additive effect was obtained also when using wt CCL5 in double combinations with the inhibitors CV-N, FTC, TDF and IDV (Fig. 3h–k and Table 3), while its combination with MVC has been already reported^42^. Considering the low expression level of the most potent CCL5 mutants produced in *L*. *jensenii*, wt CCL5 and C1C5 RANTES were the only two proteins purified to homogeneity^11^. As a proof of principle, we also verified that lactobacilli-produced CCL5 may act in combination with other HIV-1 entry inhibitors. As reported in the dose-response curves of Fig. 3l–o and in the CI_50_ of Table 3, a synergic or additive effect was observed when wt CCL5 and C1C5 RANTES were mixed in double combinations with CV-N and MVC. Considering the above proof of principle and the extremely high potency of CCL5 5p12 5 m, a combination between a live (*e*.*g*., *L*. *jensenii* secreting CCL5 5p12 5 m) and a classic microbicide could be conceived.

**Table 3:** Combination index and effect of HIV-1 inhibitor combinations.

CCL5 5p12 5 m	CI_50_	CI_75_	CI_90_	CI_50_ effect
CV-N	0.72 ± 0.04	0.72 ± 0.02	0.72 ± 0.01	Synergy
MVC	0.78 ± 0.07	0.80 ± 0.03	0.83 ± 0.01	Synergy
FTC	0.74 ± 0.08	0.81 ± 0.02	0.90 ± 0.04	Synergy
TFD	0.89 ± 0.08	0.85 ± 0.02	0.82 ± 0.04	Synergy
IDV	0.79 ± 0.12	0.79 ± 0.11	0.80 ± 0.01	Synergy
TDF + IDV	1.16 ± 0.04	1.10 ± 0.04	1.05 ± 0.01	Additivity
R4.0 + MVC	0.90 ± 0.03	0.85 ± 0.02	0.81 ± 0.06	Additivity
**wt CCL5**				
CV-N	0.98 ± 0.07	1.00 ± 0.01	1.05 ± 0.01	Additivity
FTC	0.63 ± 0.01	0.59 ± 0.01	0.56 ± 0.02	Synergy
TDF	0.16 ± 0.03	0.22 ± 0.05	0.31 ± 0.06	Synergy
IDV	0.54 ± 0.02	0.74 ± 0.01	1.03 ± 0.06	Synergy
CV-N (*L. *jensenii*)	0.96 ± 0.05	0.92 ± 0.04	0.92 ± 0.02	Additivity
MVC (*L. *jensenii*)	0.72 ± 0.02	0.69 ± 0.01	0.65 ± 0.04	Synergy
**C1C5 RANTES**				
CV-N (*L. *jensenii*)	0.93 ± 0.05	0.94 ± 0.01	0.98 ± 0.02	Additivity
MVC (*L. *jensenii*)	0.81 ± 0.07	0.80 ± 0.01	0.81 ± 0.09	Synergy

^*^CCL5 derivatives where purified from *E*. *coli*, except where specified (*L*. *jensenii*).

### CCL5 mutants interaction with CCR5: agonism *vs* antagonism

**Figure 4:**
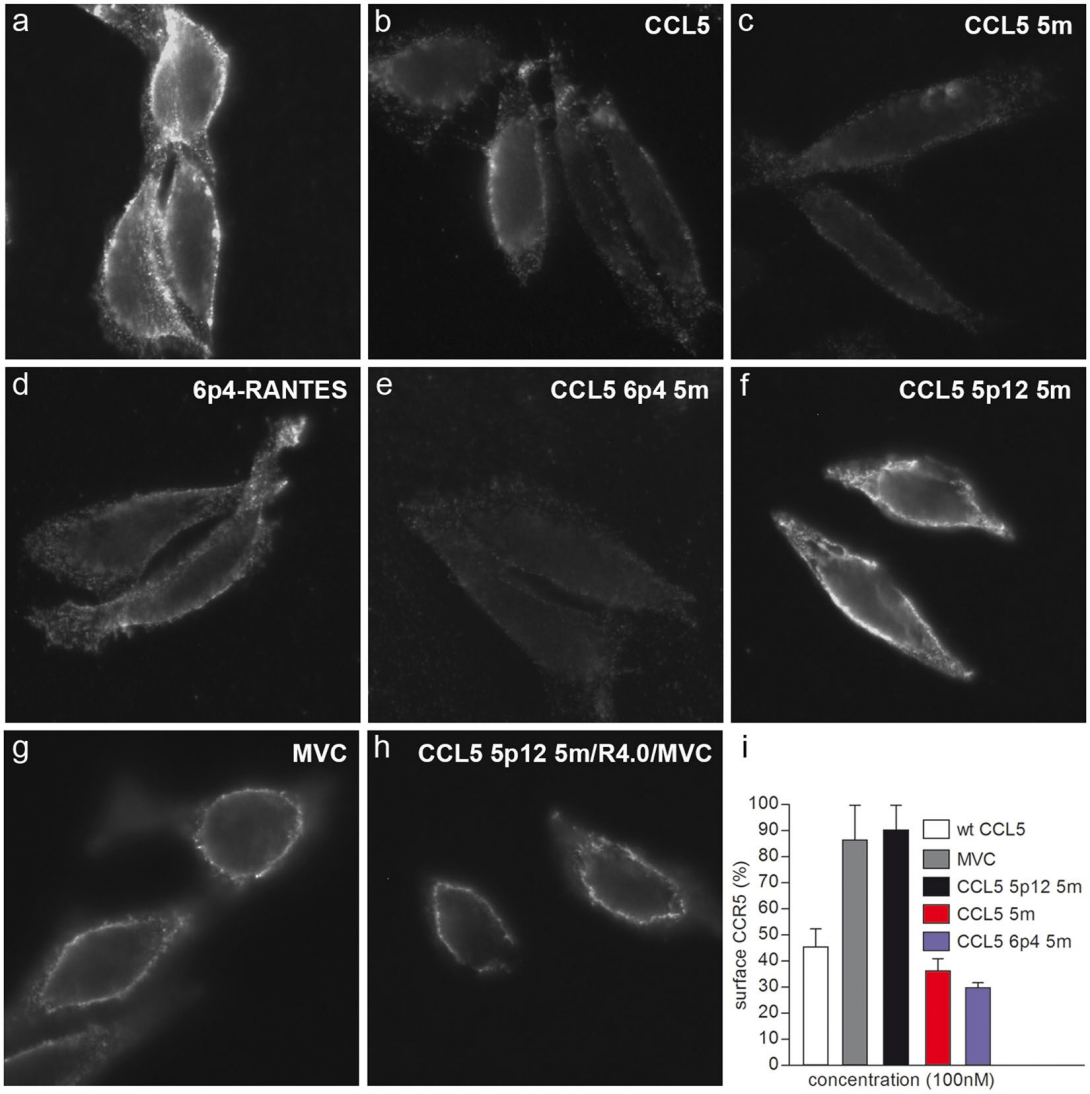
CCL5 mutants agonism or antagonism determination. (**a–h**) Immunofluorescence of CHO-CD4-CCR5 cells labeled with the anti-CCR5 mAb 3A9 (**a**), or pre-incubated with 100 nM of CCL5 derivatives: CCL5 (**b**), CCL5 5 m (**c**), 6p4-RANTES (**d**), CCL5 6p4 5 m (**e**), CCL5 5p12 5 m (**f**), MVC (**g**), CCL5 5p12 5 m, R4.0 and MVC (**h**) and labeled with 3A9. (**i**) CHO-CD4-CCR5 cell surface CCR5 measured by flow cytometry was set at 100% for cells treated with 3A9. Surface CCR5 of cells pre-incubated with 100 nM of CCL5, CCL5 5 m, CCL5 6p4 5 m, CCL5 5p12 5 m and MVC is shown as the mean ± SD of cell surface CCR5 expressed as percentage of control and is representative of two independent experiments.

CCL5 mutants were tested for their ability to induce cellular sequestration of CCR5. The need for HIV-1 blockers to interact with CCR5 without receptor internalization (antagonism) is an essential requisite in order to avoid undesirable inflammatory effects possibly promoted by chronic activation of CCR5 (agonism). Therefore, CCR5 agonism or antagonism of CCL5-derived mutants was verified by immunofluorescence using CHO-CD4-CCR5 cells and CCR5 cell surface expression was revealed by the anti-CCR5 mAb 3A9^42^. As shown in Fig. 4a–g, the introduction of our five strategic mutations did not modify the CCR5 agonism or antagonism of the respective N-terminally modified variants. CCR5 cell surface decrease was comparable when cells were treated with wt CCL5, CCL5 5 m, 6p4-RANTES and CCL5 6p4 5 m. Conversely, CCR5 antagonism (no CCR5 internalization) was observed when cells were treated with CCL5 5p12 5 m and MVC. Moreover, the triple CCL5 5p12 5 m:R4.0:MVC combination, previously tested in anti-HIV-1 acute infection (Fig. 3g), maintained a CCR5 antagonistic mode of action (Fig. 4h). CCR5 surface expression was also quantified by flow cytometry, confirming that CCR5 was not internalized when cells were treated with CCL5 5p12 5 m and MVC, while the wt CCL5, CCL5 5 m and CCL5 6p4 5 m internalized 55%, 63.5% and 70% of surface CCR5, respectively (Fig. 4i).

### Total chemical synthesis of CCL5 variants

**Figure 5:**
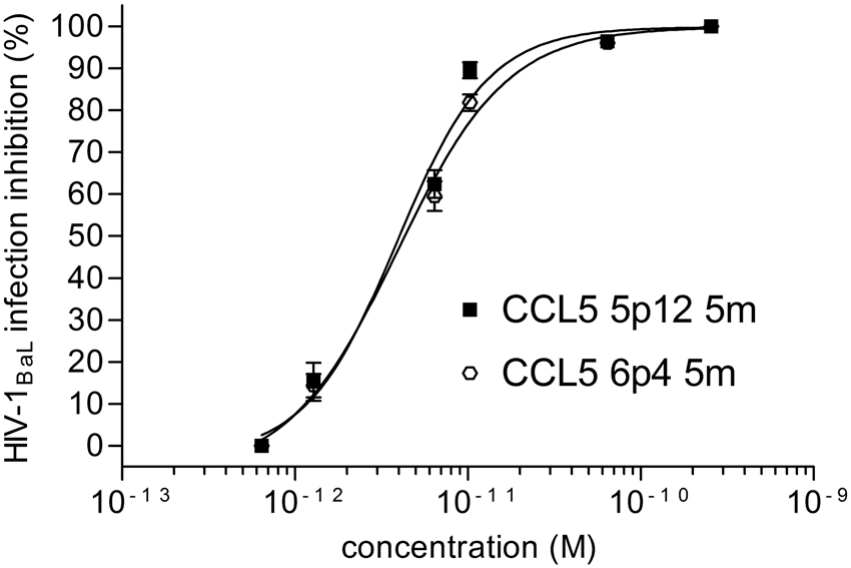
Anti-HIV-1 activity of CCL5 5p12 5 m and CCL5 6p4 5 m produced by total chemical synthesis. HIV-1_BaL_ inhibition was evaluated by acute infection assays as described in Fig. 2b. Values indicate the means ± SD of two independent experiments performed in triplicate.

CCL5 5 m, CCL5 5p12 5 m and CCL5 6p4 5 m were also produced in milligrams quantity by total chemical synthesis. When tested in acute infection assays against HIV-1_BaL_, the antiviral potency was comparable to that observed for the same CCL5 variants expressed in *L*. *jensenii* and *E*. *coli*. As shown in Fig. 5, CCL5 5p12 5 m and CCL5 6p4 5 m presented identical IC_50_s of 3.9 pM. This result is particularly interesting as it fully corroborates a parallel between chemistry and synthetic biology.

## Conclusion

Since its discovery as a natural anti-HIV-1 factor^43^, CCL5 represented a template for the engineering of potent anti-HIV-1 leads targeting CCR5^5,6^. Here, we envisaged a novel design and selection approach in which commensal lactobacilli were used as a protein expression system and a live microbicide platform to test conceptually novel CCL5 modifications. CCL5 engineering was aimed at specific hotspots, each tackled to enhance or modify the original feature, ultimately improving CCR5 binding and anti-HIV-1 activity. Through cycles of iterative mutagenesis-expression-anti-HIV-1 testing, those mutations that provided a gain of function were progressively added by stepwise insertion into CCL5 and the least promising modifications were progressively abandoned. Out of five different N-terminally-modified CCL5 variants tested, we focused on the N-terminally unmodified CCL5, the 5p12 and the 6p4 versions. The oligomerization-disrupting E66S mutation was integrated in all relevant variants, since it consistently presented an increase in anti-HIV-1 activity. Two hydrophobic regions distal to the N-terminus, identified as anti-HIV-1 activity hotspots in short CCL5-derived peptides^28–31^, have been successfully targeted on amino acids 12 (F12Y), 13 (A13V), 27 (Y27W) and 28 (F28W), and these mutations have been integrated in all relevant variants. Y27W and F28W reflected a similar anti-HIV-1 improvement observed on CCL5-derived peptides^31^, while the mutations F12Y and A13V provided a dual advantage, a decrease in lactobacilli-induced CCL5 proteolysis and an increase in anti-HIV-1 potency.

In conclusion, our work enriched the anti-HIV-1 arsenal with the most potent entry inhibitors described to date, CCL5 5p12 5 m (CCR5 antagonist) and CCL5 6p4 5 m (CCR5 agonist) and CCL5 5 m, an N-terminally unmodified CCR5 agonist of potency comparable to PSC-RANTES and 6p4-RANTES. Each lactobacilli-expressed CCL5 mutant generated here is prone to live microbicide development, a relevant option in protein-based HIV-1 prophylaxis^18,44^. Interestingly, the prospective clinical use of these new CCL5 variants is reinforced by the efforts made on 5p12-RANTES as vaginal and rectal microbicide^45,46^. In addition to the commitment to combat HIV-1 *via* systemic therapy and pre-exposure prophylaxis, these CCL5 variants (available from engineered lactobacilli, recombinant *E*. *coli* and chemical synthesis) could now be endorsed as leads for those therapeutic areas where CCL5 interaction with its receptors is of crucial relevance^47,48^.

## Methods

### Construction of expression vectors

The *E*. *coli/Lactobacillus* shuttle p1063 expression vector, a modified version of pOsel175^49^, was used for protein expression in *L*. *jensenii*^10,11^ and *L*. *rhamnosus GG*. Codon-optimized L, CCL7 and 5p12 N-terminal CCL5 variants were obtained by QuikChange site-directed mutagenesis (Stratagene) from plasmid p1063-RANTES-CO using specific primers (Supplementary Table S2), as previously described for C1C5 RANTES^11^. As indicated in Supplementary Table S2, sometimes two steps of mutagenesis were necessary to modify the N-terminal amino acids of CCL5 and obtain the final vector for the expression of the codon-optimized proteins. Instead, 6p4-RANTES was created with dedicated primers from p1063–5p12-RANTES. Then, the reported primers for E66S, T7K, Y27W, F28W and Y27W/F28W were used to introduce the corresponding mutations on the backbone of wt CCL5, L-RANTES, the CCL7/CCL5 chimera and 5p12-RANTES. The plasmid p1063-C1C5 RANTES-CO^11^ was used for the generation of C1C5 RANTES mutants. Next, mutants in positions 12 and 13 were generated on the wt CCL5 scaffold and the selected mutations F12Y, A13V, together with Y27W, F28W and E66S were introduced in the wt CCL5 and 5p12-RANTES backbones using consecutive mutagenesis cycles with the specific primers. Thus, p1063-CCL5 4 m, p1063-CCL5 5 m and p1063-CCL5 5p12 5 m were obtained. Then, p1063-CCL5 6p4 5 m was quickly created by site-directed mutagenesis of p1063-CCL5 5p12 5 m using specific primers that modified the N-terminal amino acids.

The CCL5, CCL5 5 m, 5p12-RANTES and CCL5 5p12 5 m genes for protein expression and purification in *E*. *coli* were obtained by the amplification of the nucleotide sequences from the corresponding p1063 plasmids using AmpliTaq DNA polymerase (Applied Biosystems) and specific primers (Supplementary Table S3). The resulting PCR products were inserted into pET SUMO vector following manufacturer’s instructions (Champion pET SUMO protein expression system, Invitrogen) to obtain the pET SUMO-CCL5, pET SUMO-CCL5 5 m, pET SUMO-5p12-RANTES and pET SUMO-CCL5 5p12 5 m. The pET SUMO-6p4-RANTES and pET SUMO-CCL5 6p4 5 m plasmids were obtained by site-directed mutagenesis using specific primers from pET SUMO-5p12-RANTES and pET SUMO-CCL5 5p12 5 m, respectively.

### Bacterial and viral strains

Expansion of p1063 constructs was carried out using *E*. *coli* XL1-Blue in LB broth supplemented with erythromycin (300 µg/ml). The human vaginal isolate *L*. *jensenii* 1153 (named here *L*. *jensenii* for simplicity) and the human intestinal isolate *L*. *rhamnosus GG* were routinely cultivated at 37 °C (5% CO_2_) in MRS or Rogosa SL broth, transformed with purified plasmids by electroporation, essentially as described previously^10,11^, and routinely propagated in liquid medium containing 20 µg/ml erythromycin. pET SUMO plasmids were constructed and expanded in *E*. *coli* Mach1-T1 and transformed in *E*. *coli* BL21 (DE3) for protein expression and purification.

R5 HIV-1_BaL_ was obtained from the NIH Reagent Program. R5 HIV-1 primary isolates 5513 (clade B) and 98IN007 (clade C) were kindly provided by Gabriella Scarlatti. HIV-1 isolates were propagated in phytohemagglutinin-stimulated PBMCs and the culture supernatants were harvested and stored at −80 °C until use.

### Drugs supply and formulation

MVC (Pfizer) and tenofovir disoproxil fumarate (TDF) (GILEAD) tablets (150 mg and 300 mg, respectively) and emtricitabine (FTC) powder (200 mg) contained in a capsule (GILEAD) were dissolved in sterile water and used for *in vitro* assays as described^42^. R4.0 peptide used in triple combination experiments was obtained by chemical synthesis^42^. IDV (catalog number ARP 972) was obtained from the Centre for AIDS Reagents, NIBSC, United Kingdom. CV-N was kindly provided from Osel Inc., Mountain View, CA, USA. Total chemical synthesis of CCL5 5 m, CCL5 5p12 5 m and CCL5 6p4 5 m was performed by Bachem Switzerland; 0.2 mg of the preparations were resuspended in 20 mM phosphate buffer pH 7.2, diluted and tested for anti-HIV-1 activity.

### HIV-1 infection assay

Acute HIV-1 infection was obtained by adding HIV-1_BaL_ and the primary isolates 5513 and 98IN007 (50 50% tissue culture infective doses, TCID50/well) to PM1 cells^50^ (2 × 10^4^/well) in complete RPMI medium. Experiments were performed in triplicate using 96-well round-bottom microtiter plates in the presence or absence of inhibitors and virus replication was assayed at days 4 post-infection by the p24 antigen ELISA as previously described^42^.

Human monocyte cultures were established from peripheral blood mononuclear cells (PBMC) isolated from Ficoll-Hypaque (Pharmacia) density gradient centrifugation of buffy coat preparations obtained from healthy HIV-1-seronegative blood donors. The Institutional review board named “Comitato Etico della Fondazione San Raffaele del Monte Tabor, Milan, Italy” approved the investigations. Protocol no 95/DG. All anonymized subject provided the written informed consent and all methods were performed in accordance with the relevant Italian guidelines and regulations. MDM were obtained after 7–10 days of monocyte differentiation and infected in quadruplicate with HIV-1_BaL_ (50 TCID50/well) in the presence or absence of inhibitors as described^42^. After overnight incubation, unbound virus was removed by extensive washing, fresh medium was added, and cultures were further incubated at 37 °C. Supernatants were harvested at day 4 for p24 antigen determination.

### Combinations and statistical analysis

Experimental design and analysis of synergy, additivity or antagonism between different compounds were based on the combination index (CI) method of Chou and Talalay^51,52^. In HIV-1 infection assays, each compound was tested individually and in a fixed molar ratio (IC_50_:IC_50_) combination over a range of two fold serial dilutions. The 50%, 75%, and 90% combination indexes (CI_50_, CI_75_, and CI_90_) to determine the effect of the interactions between the compounds were calculated using the Calcusyn software 2.0 (Biosoft)^42^. A CI of <0.9 indicates synergy, a CI from 0.9 to 1.1 indicates additivity, and a CI of >1.1 indicates antagonism. Dose-response curves were fitted using GraphPad Prism version 5.04 (GraphPad Software) in order to calculate IC_50_ concentrations through nonlinear regression analysis. All data are expressed as the means ± SD for two independent experiments performed in triplicate. All p-values were combined according to the Fisher’s method.

### Immunofluorescence microscopy and cytofluorimetry analysis

For immunofluorescence microscopy, 1 × 10^5^ CHO-CD4-CCR5 cells were grown in 12-multiwell on 18 mm glass coverslips (Zeus super) in complete DMEM medium^42^. One day after, cells were washed twice in PBS and incubated at 37 °C for 4 h in DMEM without FBS in the presence of 100 nM CCL5, 100 nM MVC and 100 nM CCL5 derivatives. In triple combination experiments, MVC, R4.0 and CCL5 5p12 5 m were added at the concentration of 100 nM each. Then, cells were fixed and stained with 5 μg/ml 3A9 anti-CCR5 antibody (BD Bioscience) and 2 μg/ml donkey anti-mouse IgG (H + L) Alexa Fluor 488 (Invitrogen) as described^42^. Finally, the coverslips were mounted over MOWIOL 4–88 (Sigma) and examined using a Zeiss Axiophot epifluorescence microscope (Carl Zeiss Microscopy LLC) under a 63×/1.40 oil immersion objective equipped with a Hamamatsu digital CCD camera C4742–95 (Hamamatsu Photonics). Briefly, CHO-CD4-CCR5 cells were established by CHO-K1 cells transfection with a pCDNA3.1 plasmid containing the human CCR5 gene. A CHO clone expressing CCR5 was transfected with a pCDNA3.1 plasmid containing the human CD4 gene. Stable CHO clones were analyzed by FACS to verify the surface co-expression of CCR5 and CD4.

Cytofluorimetry analysis of surface CCR5 was conducted on 1 × 10^5^ CHO-CD4-CCR5 cells incubated 4 h at 37 °C in DMEM without FBS in the presence of wt CCL5, MVC or the CCL5 variants (100 nM each). After the treatment, cells were washed twice with cold PBS containing 2% FBS and fixed with 2% fresh formaldehyde (Sigma) for 15 min. Then, washed cells were incubated with the same primary and secondary antibodies used in immunofluorescence for 15 min at 4 °C. After washing, cells were analyzed using the Gallios Flow Cytometer (Beckman Coulter Inc.) and data analyzed using the FlowJo software (Tree Star Inc.).

### Protein purification

In the anti-HIV-1 activity screening system, CCL5 derivatives were semi-purified from 50 ml of transformed *L*. *jensenii* overnight Rogosa cultures, as previously described^10^. Briefly, culture supernatants were clarified, filtered and sequentially passed through Q-Sepharose Fast Flow (FF) resin (Amersham) and SP-Sepharose FF resin (Amersham) at pH 6.0. Semi-purified proteins were eluted with a two-step NaCl gradient 300 mM - 1 M. Then, 1 M NaCl fractions containing CCL5 variants were quantified and used in the previously described p24-based assay. Purified CCL5 and C1C5 RANTES from *L*. *jensenii* were obtained as described^11^. CCL5 derivatives N-terminally linked with SUMO tag were purified from 1 liter of *E*. *coli* BL21 (DE3) as described elsewhere^34^. Briefly, 1 liter of transformed *E*. *coli* cultures were induced with 1 mM IPTG at 37 °C overnight and, after inclusion bodies solubilization, proteins were refolded overnight with 90 ml of folding buffer (50 mM NaCl, 20 mM Tris, pH 8.0) supplemented with 10 mM β-mercaptoethanol at 4 °C. Then, centrifuged supernatants were dialyzed against 4 liters of folding buffer and passed through 2 ml Ni-NTA affinity resin (Qiagen). Proteins were eluted with imidazole gradient (from 20 to 200 mM) in folding buffer and protein concentration evaluated by 13% SDS-PAGE stained with Coomassie brillant Blue R-250 (Applichem). SUMO tagged purified proteins were dialyzed in 4 liters of 50 mM NaCl, 20 mM Tris buffer, pH 7.2, to remove imidazole and the SUMO tag was removed by 2 h incubation at 37 °C with recombinant yeast ULP1 protease. ULP1 protease was produced and purified in our laboratory as described in Zhao *et al*.^34^. Purified CCL5 variants were separated from the SUMO tag using 0.5 ml Ni-NTA resin and collected in the flow through for protein quantification.

### CCL5 quantification

CCL5 variants concentration was evaluated by human CCL5/RANTES ELISA (DuoSet R&D Systems), by 13% SDS-PAGE stained with Coomassie brillant Blue R-250 and by Western blot analysis. Known concentrations of CCL5 standard (Serono) were used as reference. Western blot analysis was performed using Protran-83 nitrocellulose membranes (Schleicher & Schuell) incubated overnight with polyclonal rabbit anti-human CCL5 antibodies (1:1000) (PeproTech), followed by 1 h incubation with horseradish peroxidase-conjugated polyclonal goat anti-rabbit antibodies (1:5000) (Sigma). Chemiluminescent signals were developed using the ECL reagent (GE Healthcare Amersham).

## Electronic supplementary material

Supplementary Material
