# Supplementary material for: Rational CCL5 mutagenesis integration in a lactobacilli platform generates extremely potent HIV-1 blockers
Source: Sci Rep. 2018 Jan 30;8:1890. doi: 10.1038/s41598-018-20300-9 (PMC5790001; doi:10.1038/s41598-018-20300-9)
Supplement: Supplementary file 1 — Supplementary Material [file 41598_2018_20300_MOESM1_ESM.pdf]

Rational CCL5 mutagenesis integration in a lactobacilli platform generates extremely potent HIV-1 blockers

Massimiliano Secchi, Valentina Grampa & Luca Vangelista

Supplementary Figure S1. Linear sequence representation of wt CCL5 and CCL5 5m. Upper sequence, wt CCL5 with the five positions (F12, A13, Y27, F28 and E66) highlighted in bold. Lower sequence, CCL5 5m with the five positions (mutated to Y, V, W, W and S, respectively) highlighted in bold red.

1 10 20 30 40 50 60

SPYSSDTTPCC**FA**YIARPLPRAHIKE**YF**YTSGKCSNPAVVFVTRKNRQVCANPEKKWVREYINSL**E**MS

SPYSSDTTPCC**YV**YIARPLPRAHIKE**WW**YTSGKCSNPAVVFVTRKNRQVCANPEKKWVREYINSL**S**MS

Supplementary Figure S2. Gel image for Fig. 1B, lanes 1 and 2. Corresponding lanes in complete gel, 4 and 5.

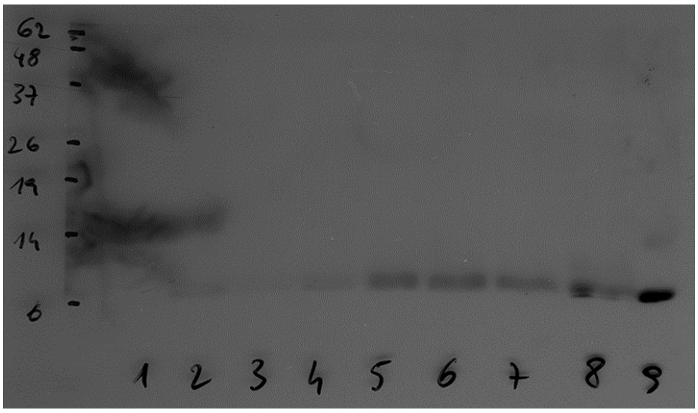

Supplementary Figure S3. Gel image for Fig. 1B, lanes 3 and 4. Corresponding lanes in complete gel, 4 and 5.

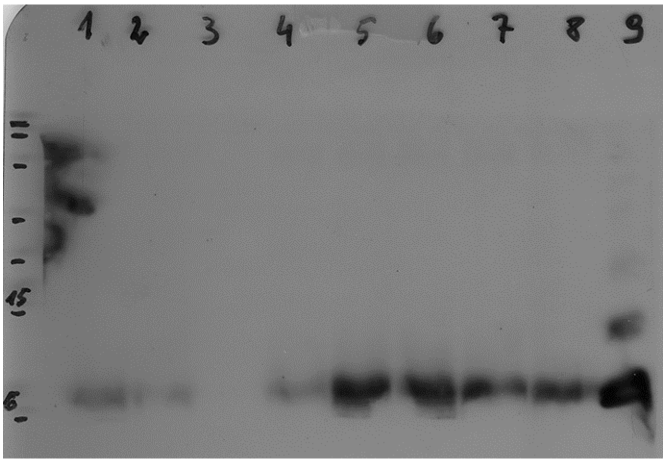

Supplementary Figure S4. Gel image for Fig. 1C. Corresponding lanes in complete gel, 1 to 6.

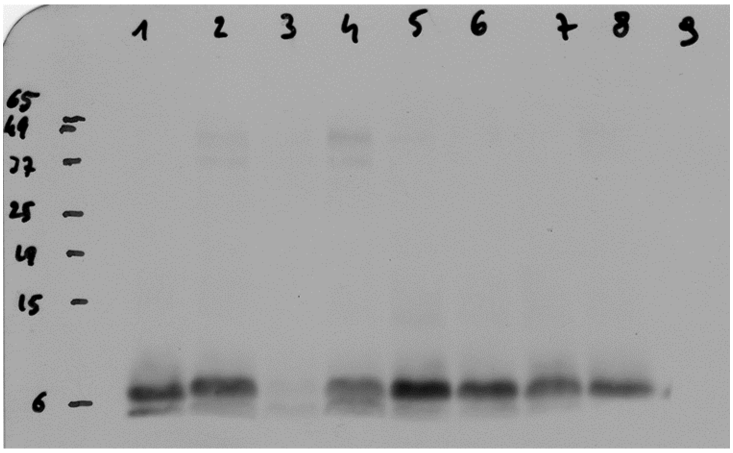

Supplementary Figure S5. Gel image for Fig. 2A, lanes 1 to 6. Corresponding lanes in complete gel, 2 to 7.

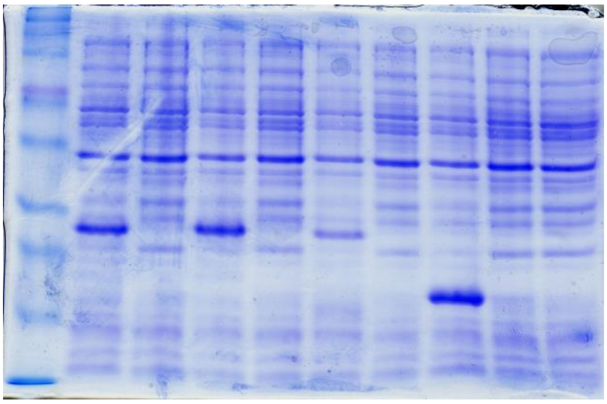

Supplementary Figure S6. Gel image for Fig. 2A, lanes 7 to 9. Corresponding lanes in complete gel, 2 to 4.

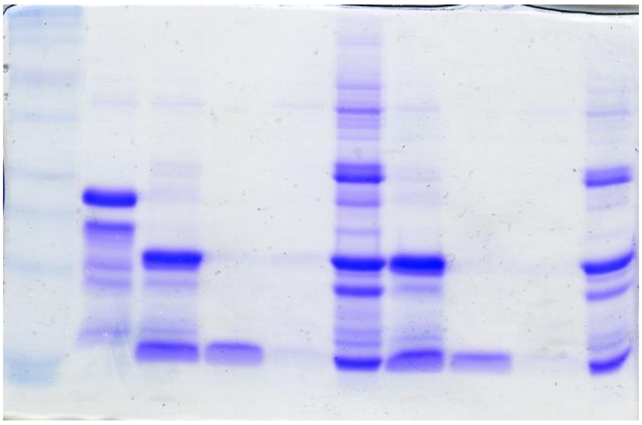

Supplementary Figure S7. Gel image for Fig. 2A, lane 10. Corresponding lane in complete gel, 4.

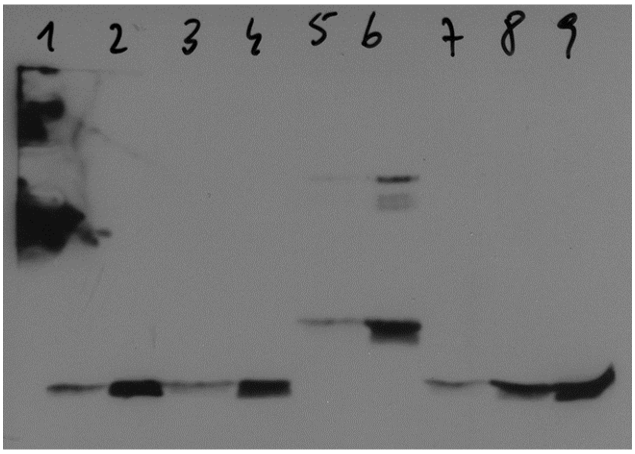

Supplementary Table S1. CCL5 mutant list (in bold red the mutated amino acids).

Mutant	Sequence	Ref.
wt CCL5	S^{1}PYSSDTTPCCFAYIARPLPRAHIKEYFYT^{30}--	5
CCL5 E66S	S^{1}PYSSDTTPCCFAYIARPLPRAHIKEYFYT^{30}--**S**^{66}	22
CCL5 T7K	S^{1}PYSSD**K**TPCCFAYIARPLPRAHIKEYFYT^{30}--	this work
CCL5 T7K/E66S	S^{1}PYSSD**K**TPCCFAYIARPLPRAHIKEYFYT^{30}--**S**^{66}	this work
CCL5 Y27W	S^{1}PYSSDTTPCCFAYIARPLPRAHIKE**W**FYT^{30}--	this work
CCL5 F28W	S^{1}PYSSDTTPCCFAYIARPLPRAHIKEY**W**YT^{30}--	this work
CCL5 Y27W/F28W	S^{1}PYSSDTTPCCFAYIARPLPRAHIKE**WW**YT^{30}--	this work
CCL5 Y27W/F28W/E66S	S^{1}PYSSDTTPCCFAYIARPLPRAHIKE**WW**YT^{30}--**S**^{66}	this work
CCL5 F12W	S^{1}PYSSDTTPCC**W**AYIARPLPRAHIKEYFYT^{30}--	this work
CCL5 F12Y	S^{1}PYSSDTTPCC**Y**AYIARPLPRAHIKEYFYT^{30}--	this work
CCL5 F12P	S^{1}PYSSDTTPCC**P**AYIARPLPRAHIKEYFYT^{30}--	this work
CCL5 A13V	S^{1}PYSSDTTPCCF**V**YIARPLPRAHIKEYFYT^{30}--	this work
CCL5 A13L	S^{1}PYSSDTTPCCF**L**YIARPLPRAHIKEYFYT^{30}--	this work
CCL5 A13M	S^{1}PYSSDTTPCCF**M**YIARPLPRAHIKEYFYT^{30}--	this work
CCL5 A13P	S^{1}PYSSDTTPCCF**P**YIARPLPRAHIKEYFYT^{30}--	this work
CCL5 A13F	S^{1}PYSSDTTPCCF**F**YIARPLPRAHIKEYFYT^{30}--	this work
CCL5 A13Y	S^{1}PYSSDTTPCCF**Y**YIARPLPRAHIKEYFYT^{30}--	this work
CCL5 A13W	S^{1}PYSSDTTPCCF**W**YIARPLPRAHIKEYFYT^{30}--	this work
CCL5 F12Y/A13V	S^{1}PYSSDTTPCC**YV**YIARPLPRAHIKEYFYT^{30}--	this work
CCL5 4m	S^{1}PYSSDTTPCC**YV**YIARPLPRAHIKE**WW**YT^{30}--	this work
CCL5 5m	S^{1}PYSSDTTPCC**YV**YIARPLPRAHIKE**WW**YT^{30}--**S**^{66}	this work
C1C5 RANTES	**C**^{1}PYS**C**DTTPCCFAYIARPLPRAHIKEYFYT^{30}--	9
C1C5 E66S	**C**^{1}PYS**C**DTTPCCFAYIARPLPRAHIKEYFYT^{30}--**S**^{66}	this work
C1C5 Y27W	**C**^{1}PYS**C**DTTPCCFAYIARPLPRAHIKE**W**FYT^{30}--	this work
C1C5 27W/28W	**C**^{1}PYS**C**DTTPCCFAYIARPLPRAHIKE**WW**YT^{30}--	this work
C1C5 Y27W/F28W/E66S	**C**^{1}PYS**C**DTTPCCFAYIARPLPRAHIKE**WW**YT^{30}--**S**^{66}	this work
L-RANTES	**L**S^{1}PYSSDTTPCCFAYIARPLPRAHIKEYFYT^{30}--	9
L E66S	**L**S^{1}PYSSDTTPCCFAYIARPLPRAHIKEYFYT^{30}--**S**^{66}	this work
L Y27W	**L**S^{1}PYSSDTTPCCFAYIARPLPRAHIKE**W**FYT^{30}--	this work
L Y27W/F28W	**L**S^{1}PYSSDTTPCCFAYIARPLPRAHIKE**WW**YT^{30}--	this work
L Y27W/F28W/E66S	**L**S^{1}PYSSDTTPCCFAYIARPLPRAHIKE**WW**YT^{30}--**S**^{66}	this work
CCL7/CCL5	**QP**^{1}**VGINTSTT**CCFAYIARPLPRAHIKEYFYT^{30}--	this work
CCL7/CCL5 E66S	**QP**^{1}**VGINTSTT**CCFAYIARPLPRAHIKEYFYT^{30}--**S**^{66}	this work
CCL7/CCL5 Y27W	**QP**^{1}**VGINTSTT**CCFAYIARPLPRAHIKE**W**FYT^{30}--	this work
CCL7/CCL5 Y27W/F28W	**QP**^{1}**VGINTSTT**CCFAYIARPLPRAHIKE**WW**YT^{30}--	this work
CCL7/CCL5 Y27W/F28W/E66S	**QP**^{1}**VGINTSTT**CCFAYIARPLPRAHIKE**WW**YT^{30}--**S**^{66}	this work
5p12-RANTES	**QG**^{1}**PPLMATQS**CCFAYIARPLPRAHIKEYFYT^{30}--	12
5p12 E66S	**QG**^{1}**PPLMATQS**CCFAYIARPLPRAHIKEYFYT^{30}--**S**^{66}	25
5p12 Y27W	**QG**^{1}**PPLMATQS**CCFAYIARPLPRAHIKE**W**FYT^{30}--	this work
5p12 Y27W/F28W	**QG**^{1}**PPLMATQS**CCFAYIARPLPRAHIKE**WW**YT^{30}--	this work
5p12 Y27W/F28W/E66S	**QG**^{1}**PPLMATQS**CCFAYIARPLPRAHIKE**WW**YT^{30}--**S**^{66}	this work
5p12 F12Y/A13V	**QG**^{1}**PPLMATQS**CC**YV**YIARPLPRAHIKEYFYT^{30}--	this work
CCL5 5p12 5m	**QG**^{1}**PPLMATQS**CC**YV**YIARPLPRAHIKE**WW**YT^{30}--**S**^{66}	this work
6p4-RANTES	**QG**^{1}**PPGDIVLA**CCFAYIARPLPRAHIKEYFYT^{30}--	12
CCL5 6p4 5m	**QG**^{1}**PPGDIVLA**CC**YV**YIARPLPRAHIKE**WW**YT^{30}--**S**^{66}	this work

Supplementary Table S2. Primers list for CCL5 mutagenesis in *L. jensenii*.

L-RANTES-fw	CTACTGTTTCAGCTTTAAGCCCTTATAGCAGTG
L-RANTES-rev	CACTGCTATAAGGGCTTAAAGCTGAAACAGTAG
Step 1 CCL7/CCL5-fw	GCCGTTTCTACTGTTTCAGCTCAACCTGTTGGTATTGATACTACTCCATGTTG
Step 1 CCL7/CCL5-rev	CAACATGGAGTAGTATCAATACCAACAGGTTGAGCTGAAACAGTAGAAACGGC
Step 2 CCL7/CCL5-fw	CAGCTCAACCTGTTGGTATTAATACTAGTACTACTTGTTGTTTTGCTTATATCGC
Step 2 CCL7/CCL5-rev	GCGATATAAGCAAAACAACAAGTAGTACTAGTATTAATACCAACAGGTTGAGCTG
Step 1 5P12-RANTES-fw	CTACTGTTTCAGCTCAAGGTCCTCCTTTAGATACTACTCCATGTTG
Step 1 5P12-RANTES-rev	CAACATGGAGTAGTATCTAAAGGAGGACCTTGAGCTGAAACAGTAG
Step 2 5P12-RANTES-fw	GCTCAAGGTCCTCCTTTAATGGCTACTCAAAGCTGTTGTTTTGCTTATATCGC
Step 2 5P12-RANTES-rev	GCGATATAAGCAAAACAACAGCTTTGAGTAGCCATTAAAGGAGGACCTTGAGC
6p4-RANTES-fw	CAGCTCAAGGTCCTCCTGGAGATATTGTTTTAGCTTGTTGTTTTGCTTATATCGC
6p4-RANTES-rev	GCGATATAAGCAAAACAACAAGCTAAAACAATATCTCCAGGAGGACCTTGAGCTG
E66S-fw	ATTAATAGTCTTAGCATGAGCTAGCAATTGTAAGC
E66S-rev	GCTTACAATTGCTAGCTCATGCTAAGACTATTAAT
T7K-fw	CCTTATAGCAGTGATAAGACTCCATGTTGTTTTGC
T7K-rev	GCAAAACAACATGGAGTCTTATCACTGCTATAAGG
Y27W-fw	GAGCTCATATTAAGGAATGGTTTTATACTAGTGGTAAG
Y27W-rev	CTTACCACTAGTATAAAACCATTCCTTAATATGAGCTC
F28W-fw	GCTCATATTAAGGAATATTGGTATACTAGTGGTAAGTG
F28W-rev	CACTTACCACTAGTATACCAATATTCCTTAATATGAGC
Y27W/F28W-fw	GAGCTCATATTAAGGAATGGTGGTATACTAGTGGTAAGTGTAG
Y27W/F28W-rev	CTACACTTACCACTAGTATACCACCATTCCTTAATATGAGCTC
F12Y-fw	CTACTCCATGTTGTTATGCTTATATCGCTAGACC
F12Y-rev	GGTCTAGCGATATAAGCATAACAACATGGAGTAG
F12W-fw	CTACTCCATGTTGTTGGGCTTATATCGCTAGACC
F12W-rev	GGTCTAGCGATATAAGCCCAACAACATGGAGTAG
A13P-fw	CTACTCCATGTTGTTTTCCTTATATCGCTAGACC
A13P-rev	GGTCTAGCGATATAAGGAAAACAACATGGAGTAG
A13F-fw	CTACTCCATGTTGTTTTTTTTATATCGCTAGACC
A13F-rev	GGTCTAGCGATATAAAAAAAACAACATGGAGTAG
A13Y-fw	CTACTCCATGTTGTTTTTATTATATCGCTAGACC
A13Y-rev	GGTCTAGCGATATAATAAAAACAACATGGAGTAG
A13V-fw	CTACTCCATGTTGTTTTGTTTATATCGCTAGACC
A13V-rev	GGTCTAGCGATATAAACAAAACAACATGGAGTAG
A13L-fw	CTACTCCATGTTGTTTTCTTTATATCGCTAGACC
A13L-rev	GGTCTAGCGATATAAAGAAAACAACATGGAGTAG
A13M-fw	CTACTCCATGTTGTTTTATGTATATCGCTAGACC
A13M-rev	GGTCTAGCGATATACATAAAACAACATGGAGTAG
A13W-fw	CTACTCCATGTTGTTTTTGGTATATCGCTAGACC
A13W-rev	GGTCTAGCGATATACCAAAAACAACATGGAGTAG
F12Y/A13V-fw for wt CCL5	GATACTACTCCATGTTGTTATGTTTATATCGCTAGACCATTACC

F12Y/A13V-rev for wt CCL5	GTAAATGGTCTAGCGATATAAACATAACAACATGGAGTAGTATC
F12Y/A13V-fw for 5p12-RANTES	GCTACTCAAAGCTGTTGTTATGTTTATATCGCTAGACCATTACC
F12Y/A13V-rev for 5p12-RANTES	GGTAATGGTCTAGCGATATAAACATAACAACAGCTTTGAGTAGC
CCL5 6p4 5m-fw	CAGCTCAAGGTCCTCCTGGAGATATTGTTTTAGCTTGTTGTTATGTTTATATCGC
CCL5 6p4 5m-rev	GCGATATAAACATAACAACAAGCTAAAACAATATCTCCAGGAGGACCTTGAGCTG

Supplementary Table S3. Primers list for CCL5 mutagenesis in *E. coli*.

wt CCL5-fw<br>(also for CCL5 5m)	AGCCCTTATAGCAGTGATACTACTCC
wt CCL5-rev<br>(also for CCL5 5mut)	CTAGCTCATCTCAAGACTATTAATATATTC
5p12-RANTES-fw<br>(also for CCL5 5p12 5m)	CAAGGTCCTCCTTTAATGGCTACTC
5p12-RANTES-rev<br>(also for CCL5 5p12 5m)	CTAGCTCATGCTAAGACTATTAATATATTC
6p4-RANTES-fw	GGTGGTCAAGGTCCTCCTGGAGATATTGTTTTAGCTTGTTGTTTTGCTTATATCGC
6p4-RANTES-rev	GCGATATAAGCAAAACAACAAGCTAAAACAATATCTCCAGGAGGACCTTGACCACC
CCL5 6p4 5m-fw	GGTGGTCAAGGTCCTCCTGGAGATATTGTTTTAGCTTGTTGTTATGTTTATATCGC
CCL5 6p4 5m-rev	GCGATATAAACATAACAACAAGCTAAAACAATATCTCCAGGAGGACCTTGACCACC
